# Ultrastable Interfacial Contacts Enabling Unimpeded Charge Transfer and Ion Diffusion in Flexible Lithium‐Ion Batteries

**DOI:** 10.1002/advs.202105419

**Published:** 2022-02-02

**Authors:** Ying Shi, Zhenxing Wang, Lei Wen, Songfeng Pei, Ke Chen, Hucheng Li, Hui‐Ming Cheng, Feng Li

**Affiliations:** ^1^ School of Materials Science and Engineering University of Science and Technology of China Shenyang 110016 China; ^2^ Ji Hua Laboratory Foshan Guangdong 528000 China; ^3^ Shenyang National Laboratory for Materials Science Institute of Metal Research Chinese Academy of Sciences Shenyang 110016 China; ^4^ School of Physical Science and Technology Shanghai Tech University Shanghai 201210 China; ^5^ Institute of Technology for Carbon Neutrality Shenzhen Institute of Advanced Technology Chinese Academy of Sciences Shenzhen 518055 China

**Keywords:** flexible lithium‐ion batteries, high‐speed centrifugal spraying, integrated electrodes, interfacial contact, O_2_ plasma

## Abstract

Deteriorating interfacial contact under mechanical deformation induces large cracks and high charge transfer resistance, resulting in a severe capacity fading of flexible lithium‐ion batteries (LIBs). Herein, an oxygen plasma treatment on a polymer separator combined with high‐speed centrifugal spraying to construct ultrastable interfacial contacts is reported. With the treatment, abundant hydrophilic oxygen‐containing functional groups are produced and ensure strong chemical adhesion between the separator and the active materials. With single walled carbon nanotubes (SWCNTs) sprayed onto the active materials, a dense thin film is formed as the current collector. Meanwhile, the centrifugal force caused by high‐speed rotation together with van der Waals forces under fast evaporation produces a much closer interface between the current collector and the active materials. As a result of this ultrastable interfacial interaction, the integrated electrode shows no structural failure after 5000 bending cycles with the charge‐transfer resistance as low as 35.8% and a Li‐ion diffusion coefficient nearly 19 times of the untreated electrode. Flexible LIBs assembled with these integrated electrodes show excellent structural and electrochemical stability, and can work steadily under various deformed states and repeated bending. This work provides a new technique toward rational design of electrode configuration for flexible LIBs.

## Graphical abstract

A universal strategy for fabricating integrated electrode with ultrastable interfacial contacts is proposed. Both the interface between active materials and separator and that between active materials and current collector are greatly improved, enabling unimpeded charge transfer and ion diffusion in flexible lithium‐ion batteries (LIBs). Excellent structural and electrochemical stability of the LIBs are obtained even under various deformed states.

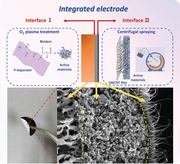

## 1 Introduction

With the dramatic development of smart, portable and wearable electronics in recent years, power sources with high energy and/or power densities, as well as flexibility are in ever‐increasing demand. Flexible power sources with good deformability and electrochemical performance are essential for wearable devices, roll‐up displays, smart textiles and bio‐sensors, which need to work under mechanical deformation, such as bending, twisting and stretching.^[^ ^1^, ^2^ ^]^ Due to their high energy density, stable cyclic stability and mature production technology, lithium‐ion batteries (LIBs) are still one of the most ideal candidates as power sources for such devices.^[^ ^3^ ^]^ A typical LIB consists of cathode, anode, electrolyte, separator and packaging. The cathodes and anodes are usually fabricated with stiff active materials coated on metal current collectors, such as copper and aluminum foils. Under repeated deformation, the active materials are easily detached from the metal current collector, leading to large cracks, high interfacial resistance, and continuous capacity loss.^[^ ^4^, ^5^ ^]^ Therefore, the development of flexible electrodes with excellent structural stability during deformation is essential for the use in flexible LIBs.^[^ ^6^, ^7^, ^8^ ^]^

For flexible electrodes, good interfacial contact between substrate and active materials is essential.^[^ ^5^ ^]^ Many efforts have been made to produce a good interfacial contact, including etching and coating the surface of current collector,^[^ ^9^ ^]^ in situ growth active materials on the substrate^[^ ^10^, ^11^, ^12^ ^]^ and vacuum filtrating active materials onto the current collector.^[^ ^13^ ^]^ The direct growth of the active materials on the metal current collector is an efficient way to construct integrated electrodes for flexible LIBs.^[^ ^14^ ^]^ However, the metal foil still suffers from limited flexibility and mechanical robustness. To resolve this problem, polymers and textiles with superior mechanical strength and light weight, such as polypropylene, polyurethane and cotton fabrics, are considered as good candidates for flexible substrates to alleviate the imposed stress under deformation.^[^ ^15^, ^16^, ^17^, ^18^ ^]^ However, the conductivity of these substrates are usually to be increased by additives, such as metal nanoparticles or nanofibers, which reduces the energy density due to the extra weight of inactive mass. With a high electrical conductivity, stable thermal and chemical properties and variety of forms, carbon materials, such as carbon nanotubes (CNTs), graphene and carbon textiles are considered as another important kind of substrate.^[^ ^19^, ^20^, ^21^, ^22^, ^23^, ^24^, ^25^ ^]^ Among them, CNTs have been widely used as a multi‐functional material in LIBs for decades. Moreover, the special one dimensional (1D) structure and high mechanical strength of CNTs make them more suitable for self‐supporting composites with active materials or directly acting as a conductive additive to construct efficient conductive networks.^[^ ^26^, ^27^, ^28^, ^29^, ^30^ ^]^ CNTs can form a segregated network in materials to suppress mechanical instability and toughening the composite as well.^[^ ^31^ ^]^ When assembled into thin films, CNTs can also serve as flexible current collectors to replace metal foils.^[^ ^32^, ^33^ ^]^ Therefore, they have great potential for the fabrication of flexible electrodes with better interfacial contact and a lower interfacial resistance. Besides the interface between active materials and current collector, the interface between the separator and active materials also needs to be considered, as ion transfer in this interface also greatly affects the electrochemical performance under deformation.

In this work, we developed an integrated electrode for flexible LIBs with ultrastable interfacial contact using an O_2_ plasma combined with high‐speed centrifugal spraying of single walled carbon nanotubes (SWNCTs) for flexible LIBs. Two essential interfaces, that between the active materials and separator, and that between the active materials and current collector, are greatly improved. With the O_2_ plasma treatment, abundant hydrophilic oxygen‐containing functional groups are generated on the surface of the separator which enables strong chemical adhesion between it and the active materials. By high‐speed centrifugal spraying, a uniform, ultrathin and strong SWCNT film to act as the current collector is directly formed on the surface of the active materials, ensuring a physically interwoven network between them. With these strategies, much closer interfacial contact is achieved and the interfacial resistance is greatly reduced, enabling a stable electrochemical performance, long cycle life and good structural stability of the integrated electrodes against thousands of bending. Therefore, the flexible LIBs assembled with these integrated electrodes can work stably in various deformed states, even after repeated bending.

## 2 Results and Discussion

### 2.1 Fabrication of Integrated Electrodes with a Sandwich Structure

**Figure 1:**
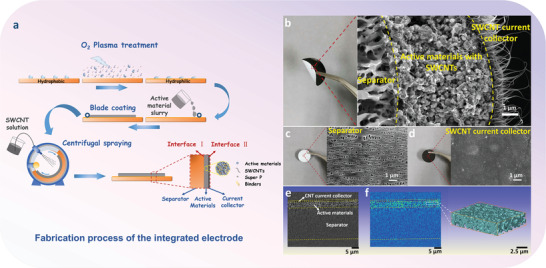
Schematic of the fabrication and structure of an integrated electrode. a) Fabrication process. Photographs and field emission scanning electron microscope (SEM) images of the integrated electrode: b) cross section, c) separator side, and d) SWCNT side. e,f) XRM images of the integrated electrode and the internal distribution of the active materials.

The fabrication of integrated electrodes is schematically shown in **Figure** **1**a. O_2_ plasma was used to treat one side of a polypropylene (PP) separator, changing its surface from hydrophobic to hydrophilic. A slurry of active materials was coated on the modified surface and, after drying, SWCNT solution was sprayed against the surface of active materials by high‐speed centrifugal spraying to directly form a film as current collector. As a result, a flexible integrated electrode with a sandwich structure was obtained, containing three parts: the separator, active material layer and SWCNT film. In the active material layer, the same SWCNTs were also used as conductive additives combined with Super P. In the sandwich structure, two important interfaces were focused on. One is between the separator and the active material, denoted Interface I, and the other is between the active material and the SWCNT film, denoted Interface II.

With this simple and universal method, many kinds of integrated electrodes can be fabricated. Here, the structure of the integrated electrodes with LiFePO_4_ (LFP) as the active material has been characterized. Photographs of the electrode from the side (Figure 1b), front (Figure 1c), and back (Figure 1d) suggest that it is intact, flat, smooth and flexible. The three parts and the two interfaces are clearly seen. The separator with many macropores was tightly connected to the active material, which facilitated the diffusion of Li ions in the electrolyte. The porous structure shown in Figure 1c is nearly the same as the untreated one (Figure S1, Supporting Information). In the inner layer of the electrode, SWCNTs were uniformly dispersed throughout the active material, forming a highly efficient conductive network. The SWCNT film fabricated using the same SWCNTs was firmly attached to the active material layer, ensuring good electronic transport. From the side of SWCNT film (Figure 1d), the active material under the SWCNT film can be indistinctly seen through the transparent current collector film. X‐ray micro‐tomography (XRM) images further confirm the three layers structure of the integrated electrode (Figure 1e). Many pores were clearly observed in the layer of active material (Figure 1f) which play an important role in the storage of the electrolyte and enable good ionic diffusion through the active material.

### 2.2 Improvement of the Two Interfaces of the Integrated Electrodes

**Figure 2:**
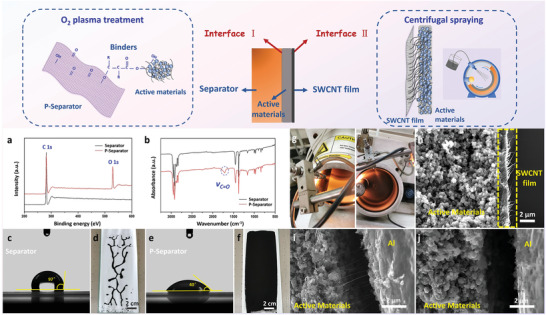
Improvement of Interface I and Interface II. a) X‐ray photoelectron spectroscopy (XPS) spectra and b) Fourier transform infrared spectroscopy (FTIR) spectra for the surface of a separator before and after plasma treatment. Contact angle measurements of the separator surface c) before and e) after plasma treatment. Photographs of the slurry coating on the separator d) before and f) after surface treatment. g) The equipment for centrifugal spraying. h) SEM images of the electrode and its interface between the active materials and the SWCNT film. SEM images of the interface between the active materials and an Al current collector i) with and j) without SWCNTs as a conductive additive in the active materials.

Interfacial adhesion is a critical factor for the long‐term performance of LIBs, especially for flexible batteries.^[^ ^34^ ^]^ As a PP separator is hydrophobic and can hardly form a good contact with the aqueous slurry of active material, the modification of its surface is necessary. In order to avoid extra inactive components, an O_2_ plasma process was chosen as a green method for the treatment of Interface I as illustrated in **Figure** **2**. This treatment can efficiently modify the polymer surface at the molecular level without damaging or changing its bulk properties^[^ ^34^, ^35^ ^]^ and generate a large number of hydrophilic oxygen‐containing functional groups, such as C═O and C–OH on its surface, giving the separator good wettability and facilitating the diffusion of the electrolyte as well.^[^ ^36^, ^37^ ^]^

A comparison of the XPS spectra of separators with and without O_2_ plasma treatment is shown in Figure 2a. A remarkable peak for O 1s at 532 eV is clearly observed for the plasma‐treated separator (P‐Separator), suggesting that oxygen functional groups had been introduced. The FTIR spectra in Figure 2b further confirm the existence of the newly formed oxygen functional groups. A strong peak at 1713 cm^–1^ for the P‐Separator is probably due to the stretching vibration of a carbonyl groups (C═O). The contact angles between water and the surface of separators before and after treatment (Figure 2c,e) also confirm these changes. After the O_2_ plasma treatment, slurry coating becomes much easier and the slurry is smoothly spread on the surface (Figure 2f), indicating better hydrophilicity of the surface. In contrast, the slurry did not spread well on the surface of the untreated separator, but shrank into a dendritic state after coating (Figure 2d). With the plasma treatment, interfacial adhesion between active materials and separator was greatly improved.

The interface between active materials and current collector usually plays an important role on the electrochemical properties. Good interfacial contact facilitates electron transport, lowers the interfacial resistance, and increases the cyclic stability.^[^ ^12^, ^18^, ^38^, ^39^ ^]^ Here, a centrifugal spraying was used to modify Interface II. By a centrifugal spraying equipment (Figure 2g), a uniformly dispersed SWCNT solution was sprayed directly onto the surface of the active materials to form a thin SWCNT film as a current collector. The heater inside the equipment quickly dried the liquid during the centrifugal spraying, giving a van der Waals contraction force under evaporation to make the SWCNT film much denser. Meanwhile, the centrifugal force produced by the high‐speed rotation tightly compressed the surface of the electrode, producing a much closer contact for Interface II.

The features of the SWCNT film were characterized by Raman spectra (Figure S2, Supporting Information) and transmission electron microscope (TEM) (Figure S3, Supporting Information). The results suggested that it is mainly composed of SWCNTs with a diameter of 1–2 nm. From the SEM image of the SWCNT film removed from a substrate (Figure S4, Supporting Information), the SWCNTs in the film are inextricably interwoven and/or intertwined together, and the film is quite continuous and intact. Many single and small bundles of SWCNTs are observed at the edge of the film. When the SWCNTs was sprayed directly onto the surface of the active materials, the film firmly adhered to it, forming a tightly interfacial contact by van der Waal forces (Figure 2h and Figure S5, Supporting Information). As control, the same electrode slurry with and without SWCNTs as a conductive additive were coated on aluminum (Al) current collectors and SEM images (Figure 2i,j) of the cross sections between active materials and Al foil showed that both electrodes have obvious gaps with a width of 2–3 µm at the interfaces. These gaps would cause bad contact and large interfacial resistance. However, for the electrode with the SWCNTs as conductive additive, the fibrous SWCNTs and bundles bridged the gaps between the active materials and the Al foil, giving a better electronical conduction than that for the electrode without SWCNTs.

### 2.3 Electrochemical Properties of Integrated Electrodes

**Figure 3:**
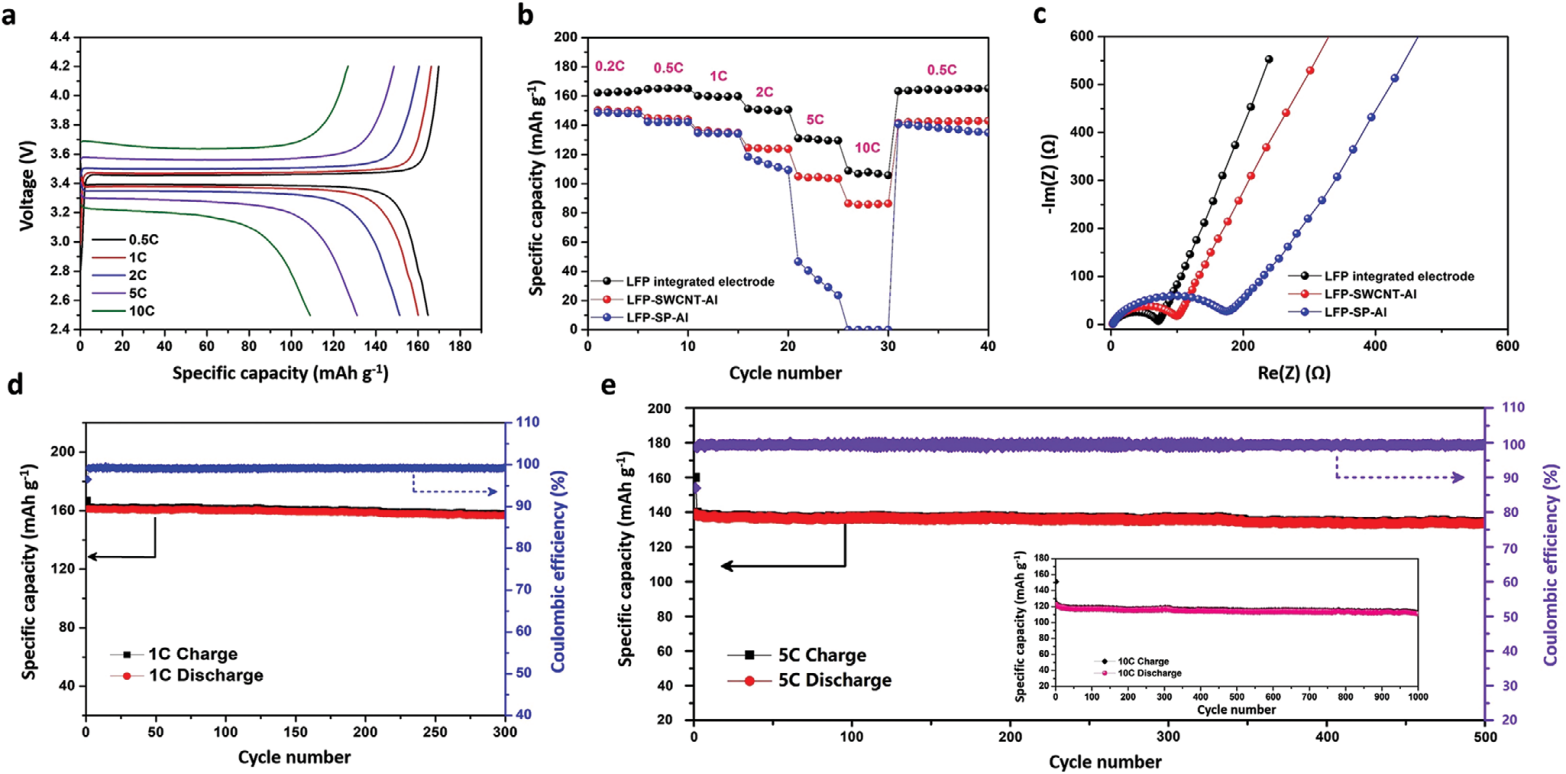
Electrochemical properties of flexible integrated electrodes. a) Charge/discharge curves at different rates. b) Rate capability and c) EIS curves of the LFP integrated electrode, LFP‐SWCNT‐Al and LFP‐SP‐Al electrodes. d,e) Long‐term cycling performance of the LFP integrated electrode at 1, 5, and 10 C rates (inset of (e)).

Charge/discharge curves at different rates for the integrated electrode with LFP as the active material were obtained in half‐cells with lithium foil as a counter electrode. **Figure** **3**a shows the typical behavior of a LFP electrode with a 3.46 V charge plateau at 0.5 C rate, with flat potential plateaus in both charge and discharge curves. The rate behavior of the LFP integrated electrode was examined from 0.5 to 10 C. Electrodes with the same active material coating on the Al foils with and without SWCNTs as a conductive additive, denoted LFP‐SWCNT‐Al and LFP‐SP‐Al, respectively, were also tested. The results in Figure 3b show that compared with the electrodes coating on the Al foils, the integrated electrode exhibits a higher specific capacity at all test rates. In view of the negligible capacity contributed by the SWCNT film in the tested potential range (Figure S6, Supporting Information), the superior rate behavior of the integrated electrode is mainly attributed to the improved interfacial contact of both Interface I and Interface II. In addition, the LFP‐SWCNT‐Al electrode has a better rate performance than the LFP‐SP‐Al electrode at high rates, from 2 to 10 C, suggesting the important effect of SWCNTs on improving the conductive connection between the active materials and the Al foil, as well as reducing the internal resistance among the active materials. With the ultrathin SWCNT film as current collector, the gravimetric energy density of the integrated electrode was also greatly improved, and superior to many other works (Table S1, Supporting Information). For the Li_4_Ti_5_O_12_ (LTO) integrated electrode, investigated as a typical anode for flexible LIBs, similar results were obtained (Figure S7, Supporting Information). Commercial cathode LiCoO_2_ (LCO) was also used to fabricate the integrated electrode in this work, and the results shown in Figure S8 (Supporting Information), confirmed the universality of this method. Electrochemical impedance spectroscopy (EIS) results shown in Figure 3c and CV curves (Figure S9, Supporting Information) confirmed the lowest charge transfer resistance and the fastest kinetics of Li^+^ insertion/deinsertion of the integrated electrode. With its improved interfacial contacts, the LFP integrated electrode also showed a stable long‐term cycling performance. A 97.2% capacity retention and an excellent retained Coulombic efficiency after 300 charge/discharge cycles at 1 C were achieved (Figure 3d). At a high rate of 5 C, a capacity retention of 96.0% and a Coulombic efficiency of 99.3% were achieved after 500 cycles (Figure 3e). When charged and discharged at the 10 C, the electrode had a capacity retention of 90.5% after 1000 cycles (inset of Figure 3e).

After long‐term charge/discharge cycling, the integrated electrode still looked like the uncycled one, with no pulverization and exfoliation. As the SWCNT film is firmly adhered to the active materials and difficult to remove, adhesive tape was used to get rid of the SWCNT film and expose the inner structure (Figure S10, Supporting Information). After long‐term cycling, both the inner structure and the cross section of the electrode had a similar morphology (Figure S11, Supporting Information) to the uncycled one, demonstrating good retention of the microstructural integrity.

### 2.4 Stability of the Flexible Integrated Electrodes

The mechanical strength and structure retention of electrodes are of great importance for flexible LIBs, because deformation can accelerate the decay of electrochemical properties. In order to measure the mechanical strength, the SWCNT film, separator and integrated electrode were stretched using a dynamic mechanical analyzer. As shown in Figure S12 (Supporting Information), the SWCNT film has good tensile strength and the separator has both higher toughness and tensile strength. As the active material layer has been sandwiched between the SWCNT film and the separator, the integrated electrode displays a good mechanical strength, much better than that of the electrode without the separator as substrate.

**Figure 4:**
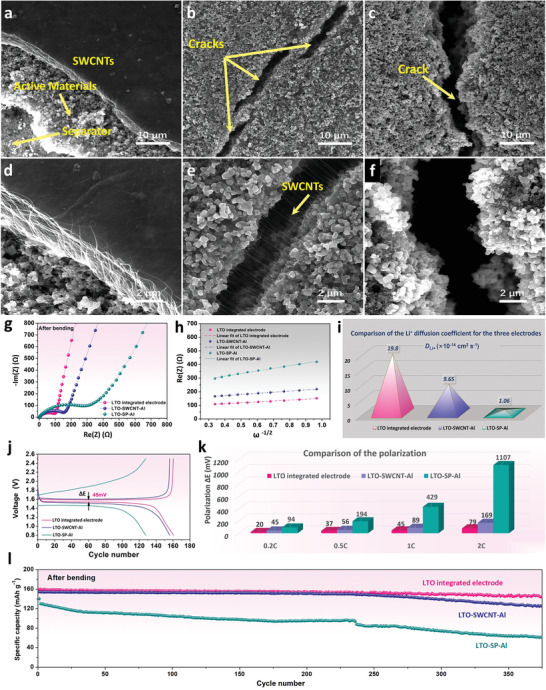
Microstructures after 5000 bending cycles of a,d) LTO integrated electrode, b,e) LTO‐SWCNT‐Al electrode, and c,f) LTO‐SP‐Al electrode. g) EIS curves, h) relationship between *Z*_re_ and *ω* ^−1/2^ in the low frequency region, and i) the comparison of the Li^+^ diffusion coefficient of the three electrodes after bending. j) Charge/discharge curves at 1 C of the three electrodes after bending and k) their polarization at different rates. l) Cycling performance of the three electrodes after bending.

To investigate the structural retention of the integrated electrode during deformation, the microstructures of a LTO electrode after 5000 bending cycles were examined by SEM. As shown in **Figure** **4**a,d, the surface of the integrated electrode was flat and intact without any cracks. After getting rid of the SWCNT film by adhesive tape, the exposed internal structure of the LTO integrated electrode still showed no cracks or breakage, and was almost the same as the structure of the electrode without bending. Electrodes with the same active material coating on the Al foils with and without SWCNTs were also investigated, denoted LTO‐SWCNT‐Al and LTO‐SP‐Al, respectively. For the LTO‐SWCNT‐Al electrode, the tiny cracks appeared on its surface (Figure 4b). However, the SWCNTs uniformly dispersed in the active materials tightly bound the materials together in the cracks (Figure 4e), therefore ensuring effective conductive bridges. Here, the special bonding effect of the SWCNTs with the 1D structure provided a better interfacial connection between the active materials as well. Indeed, this bonding effect of fibrous SWCNTs improved not only the interfaces between the active materials, but also that between active materials and current collector, which was observed in Figure 2i. When combined with the centrifugal spraying process, this effect of SWCNTs on interfacial improvement became more significant. In contrast, the LTO‐SP‐Al electrode contained large deep cracks after repeated bending (Figure 4c,f), which would block the charge transport and lengthen the ion diffusion path. To further examine the stability of these electrodes, they were immersed in the electrolyte and undergone ultrasonication for 5 min. The electrolyte with the LTO‐SP‐Al electrode is the most turbid of the three samples with many active materials detached from the electrode, while the electrolyte with integrated electrode is almost unchanged with no detached active materials, indicating its good structural stability (Figure S13, Supporting Information).

Cracking and delamination of electrodes usually produces partially isolated particles and causes poor electron/ion transport kinetics.^[^ ^5^ ^]^ Therefore, structural stability has a great effect on the electrochemical performance, especially the interfacial contact resistance. EIS measurements were carried to investigate the effect of the microstructural changes on the interfacial resistance of these electrodes. The results before and after bending are shown in Figure S14 (Supporting Information) and Figure 4g. Based on the Randles‐Ershler equivalent circuit (inset in Figure S15, Supporting Information), the charge‐transfer resistance (*R*_ct_) corresponding to the interfacial resistance between the electrolyte and electrode was deduced. For the LTO integrated electrode, the EIS curves almost remained the same before and after bending, with *R*_ct_ values of 80.6 and 85.7 Ω, respectively. For the LTO‐SWCNT‐Al electrode, *R*_ct_ changed from 135.8 to 141.0 Ω after bending, and for the LTO‐SP‐Al electrode, *R*_ct_ increased from 201.7 to 239.5 Ω, nearly 19%. The significantly increased *R*_ct_ for the LTO‐SP‐Al electrode is attributed to the poor conducting connections and large interfacial resistance produced by the cracks and breakage after repeated bending. The lowest *R*_ct_ of the integrated electrode demonstrates that its structural stability is much better and its interfacial resistance is well maintained even after thousands of bending cycles.

The effect of structural stability on Li‐ion diffusion after bending was also investigated. Based on the Nyquist plots, the Li‐ion diffusion coefficients of the three electrodes can be calculated from the inclined line in the low‐frequency region according to the following equation:^[^ ^40^, ^41^, ^42^ ^]^

(1) $${D_{\mathrm{Li}}}^{+}=R^{2}T^{2}/2A^{2}F^{4}n^{4}C^{2}\delta^{2}$$

where *D*_Li_+ is the diffusion coefficient of lithium ions, *R* the gas constant, *T* the absolute temperature, *A* the surface area of the electrode, *n* the number of transferred electrons per molecule (*n* = 1), F is Faraday's constant, *C* the concentration of Li‐ion in the electrode material, and *δ* the Warburg coefficient which is related to *Z*_re_ according to the following equation:^[^ ^42^, ^43^ ^]^

(2) $$Z_{\mathrm{re}}=R_{\mathrm{ct}}+R_{s}+\delta \omega^{-1/2}$$

where *ω* is the frequency in the low‐frequency region. Based on the fitted lines of the EIS results (Figure 4h, Table S2. Supporting Information), the Li^+^ diffusion coefficient of the three electrodes after bending can be calculated. As shown in Figure 4i, the LTO integrated electrode had the highest Li‐ion diffusivity (1.98 × 10^–13^ cm^2^ s^−1^), which was twice that of the LTO‐SWCNT‐Al electrode (9.65 × 10^–14^ cm^2^ s^−1^) and nearly 19 times that of the LTO‐SP‐Al electrode (1.06 × 10^–14^ cm^2^ s^−1^). The high Li‐ion diffusivity can be attributed to the well‐kept microstructure and the improved interfacial properties.

Benefiting from these improvements, the electrochemical performance of the electrode was also obviously enhanced. Figure 4j shows the charge and discharge curves of the three cycled electrodes at 1 C. Among them, the LTO integrated electrode displayed the smallest potential difference (∆*E*) between charge and discharge plateaus, which suggests the lowest polarization and best reaction kinetics. And this was further confirmed by the comparison of polarization at different rates shown in Figure 4k. With large and deep cracks after repeated bending, the LTO‐SP‐Al electrode displayed the biggest polarization. When the current density increased, the polarization became much more serious, the ∆*E* was nearly 14 times as much as the LTO integrated electrode at 2 C. Long‐term cycling performance of the three electrodes after bending was measured at 1 C, the results shown in Figure 4l demonstrated that with the good interfacial property, the LTO integrated electrode had the most stable cycling performance, while the LTO‐SP‐Al had the worst due to its poor conductivity and large cracks.

### 2.5 Performance of Flexible Cells with Integrated Electrodes

**Figure 5:**
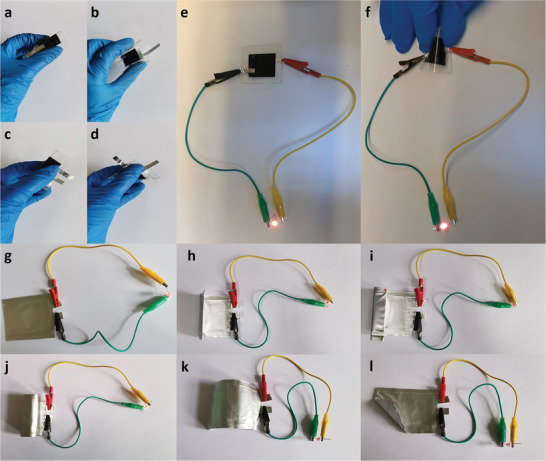
A flexible LIB assembled with integrated electrodes packaged with a–f) PDMS film and g–l) Al‐plastic film. A flexible device with PDMS film (a–d) under various bending states. After electrolyte injection and charging, the flexible LIB lit an LED in the flat and bent states (e, f). The pouch cell with Al‐plastic film under different deformation states (g, j), including flat (g), bent (h, k, l)), folded (i), and rolled state (j).

With the improved interfacial properties and good structural stability, the integrated electrodes were assembled into full cells. Full cells with LFP and LTO integrated electrodes were charged and discharged at different rates (Figure S16, Supporting Information) with very flat platforms during both processes, suggesting good electrochemical performance. Flexible cells with Poly‐(dimethyl siloxane) (PDMS) films as packaging materials were assembled, which can be easily bent to various angles (**Figure** **5**a–d). After being charged to 2.5 V, the flexible LIB easily can light an LED at both the normal and bending state (Figure 5e,f). Even under repeated bending, the flexible LIB still worked stably, as shown in Movie S1 (Supporting Information). Pouch cells packaged with an Al‐plastic film were also fabricated to further confirm the good deformability and stability at different deformation states, including flat (Figure 5g), bent (Figure 5h,k,l), folded (Figure 5i), and rolled (Figure 5j). The charge/discharge curves measured under both plat and bent states at 0.5 C were nearly the same (Figure S17, Supporting Information), demonstrating the good stability of the improved integrated electrodes. The stable electrochemical performance under different deformations indicates the great potential for the practical use of these flexible LIBs.

## 3 Conclusions

We have developed an integrated electrode with ultrastable interfacial contacts for flexible LIBs. Two crucial interfaces in the LIBs, that between the active materials and separator, and that between the active materials and current collector, have been greatly improved by the strategies of O_2_ plasma treatment of polymer separator and high‐speed centrifugal spraying of SWCNTs on active materials. SWCNTs used for both the current collector and as a conductive additive in the active materials efficiently enhance the interfacial contact and greatly reduced the interfacial resistance. With the obtained sandwich structure and the improved interfacial contact, the integrated electrodes gave a very stable electrochemical performance, long cycle life and good structural stability. Even after thousands of repeated bending cycles, the microstructure was well remained and the interfacial resistance had almost no change. As a result, flexible LIBs assembled with the integrated electrodes worked stably under various deformations, indicating their great potential for practical application. The method of forming the current collector directly on the active materials by high‐speed centrifugal spraying suggests a new way to improve the interface in other electrodes.

## 4 Experimental Section

### Preparation of the Integrated Electrode

The PP separator (Celgard 2400) was first attached to a polyethylene terephthalate (PET) film, and transferred into a plasma cleaner (Diener, Germany). After treated 60 s with an oxygen atmosphere (80 W, 0.5 NL h^–1^), the surface of the treated side was coated with the prepared slurry of active materials that contained LFP or LTO as the active materials, Super P (TIMCAL) as a conductive additive and alginic acid sodium salt (MP Biomedicals) as an aqueous binder, in weight ratios of 90:5:5. When SWCNTs (OCSiAl) were used as a conductive additive, 30% of the Super P was replaced by SWCNTs, which means the SWCNT content was 1.5 wt% of the electrode. After the slurry dried, the separator‐based electrode was transferred to a Continuous Centrifugation Coating System (ShenZhen Matterene Tech. Co. Ltd.). A uniformly dispersed SWCNT solution (0.35 mg mL^–1^) was pumped into the system and sprayed onto the surface of the active materials. The thickness of the SWCNT film was controlled by the mass or concentration of the SWCNT solution. The speed of the roller was above 2000 r min^–1^ to produce tight contact between the SWCNTs and the active material layer caused by the large centrifugal force. The as‐prepared integrated electrode was finally taken off the system and cut into disks for further characterization and electrochemical testing.

For comparison, electrodes with a direct slurry coating on the Al foils were also prepared with and without SWCNTs as conductive additive, and were denoted LFP‐SWCNT‐Al, LTO‐SWCNT‐Al, LFP‐SP‐Al and LTO‐SP‐Al.

### Fabrication of the Flexible Cells with the Integrated Electrode

Poly‐(dimethyl siloxane) (PDMS) membranes were prepared and used as a packaging material for the flexible LIBs with the integrated electrodes. Al belt was used as a tab and fixed on the edge of the integrated electrode by high‐purity conductive silver paste. Before charging, the electrolyte had to be injected into the assembled flexible device. Excess electrolyte was removed from the electrodes to ensure good sealing of the package. For the pouch cell, Al‐plastic films were used as the packaging material. A Ni belt was used as the tab welded onto the Al belt which connected with the integrated electrode.

### Characterization

The morphology and structure of the integrated electrode, separator and SWCNT film were characterized on field emission scanning electron microscope (SEM, FEI Verios 460). A Tecnai F20 high resolution transmission electron microscope (TEM) was also used for the SWCNT examination. X‐ray microtomography (XRM) of the integrated electrode was performed using a Versa XRM‐500 desktop system with an acceleration of 50 kV. X‐ray photoelectron spectroscopy (XPS) analysis was performed using an ESCALAB 250 instrument with Al *Kα* radiation (15 kV, 150 W) under 4 × 10^–8 ^Pa. Fourier transform infrared spectroscopy (FTIR) analysis was made on a Nicolet iS5 iD7 ATR spectrometer equipped with a diamond KBr beam splitter. Contact angles were measured by a contact angle meter (POWEREACH JC2000D1). Mechanical properties were measured using a dynamic mechanical analyzer (DMA Q800). Raman spectra were collected using a Jobin Yvon HR800 with a 632.8 nm excited laser.

### Electrochemical Measurements

The electrochemical performance of the integrated electrode was determined using both half and full cells, assembled in an argon‐filled glove box. For the half cell, lithium foil was used as counter electrode and 1 m LiPF_6_ in ethylene carbonate and diethyl carbonate (1:1 vol/vol) as the electrolyte. The charge/discharge curves and cycling performance were measured using a multi‐channel battery testing system (Land CT3001A) with cut‐off voltages of 2.5–4.2 V for LFP and 0.8–2.5 V for LTO, respectively. For the full cell, the operating voltage was in the range 1.0–2.5 V. The capacities and currents were calculated based on the weight of the cathode material.

## Conflict of Interest

The authors declare no conflict of interest.

## Supporting information

Supporting Information

Supplemental Movie 1

## Data Availability

The data that support the findings of this study are available from the corresponding author upon reasonable request.
