# Supplementary material for: Ultrastable Interfacial Contacts Enabling Unimpeded Charge Transfer and Ion Diffusion in Flexible Lithium‐Ion Batteries
Source: Adv Sci (Weinh). 2022 Feb 2;9(10):2105419. doi: 10.1002/advs.202105419 (PMC8981437; doi:10.1002/advs.202105419)
Supplement: Supplementary file 1 — Supporting Information [file ADVS-9-2105419-s001.pdf]

ADVANCED SCIENCE
Open Access

Supporting Information

for *Adv. Sci.*, DOI 10.1002/advs.202105419

Ultrastable Interfacial Contacts Enabling Unimpeded Charge Transfer and Ion Diffusion in Flexible Lithium-Ion Batteries

*Ying Shi, Zhenxing Wang, Lei Wen, Songfeng Pei, Ke Chen, Hucheng Li, Hui-Ming Cheng and Feng Li**

ADVANCED SCIENCE
Open Access

Supporting Information

for *Adv. Sci.,* DOI: 10.1002/advs.202105419

Ultrastable interfacial contacts enabling unimpeded charge transfer and ion diffusion in flexible lithium-ion batteries

*Ying Shi, Zhenxing Wang, Lei Wen, Songfeng Pei, Ke Chen, Hucheng Li, Hui-Ming Cheng, Feng Li**

Y. Shi, H. C. Li, Prof. F. Li

School of Materials Science and Engineering, University of Science and Technology of China, Shenyang 110016, China

Z. X. Wang

Ji Hua Laboratory, Foshan, Guangdong 528000, China

Y. Shi, Z. X. Wang, Dr. L. Wen, S. F. Pei, K. Chen, H. C. Li, Prof. H.-M. Cheng, Prof. F. Li

Shenyang National Laboratory for Materials Science, Institute of Metal Research, Chinese Academy of Sciences, Shenyang 110016, China

E-mail: fli@imr.ac.cn

K. Chen

School of Physical Science and Technology, Shanghai Tech University, Shanghai 201210, China

Prof. H.-M. Cheng

Institute of Technology for Carbon Neutrality, Shenzhen Institute of Advanced Technology, Chinese Academy of Sciences, Shenzhen 518055, China

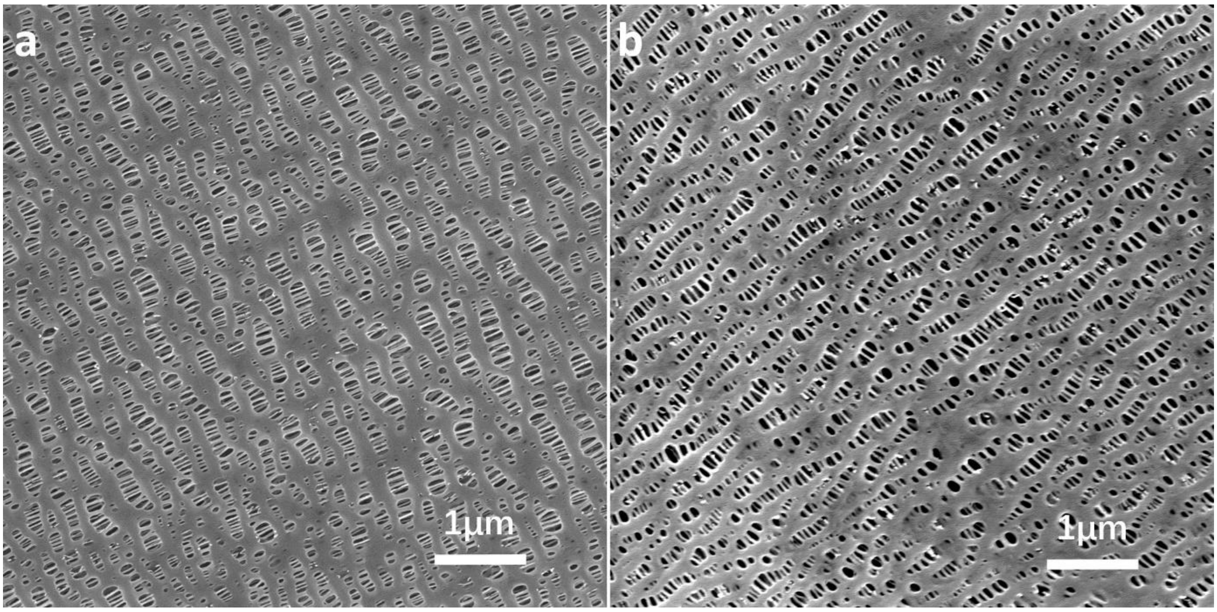

Figure S1. Morphology of the separator a) before and b) after plasma treatment.

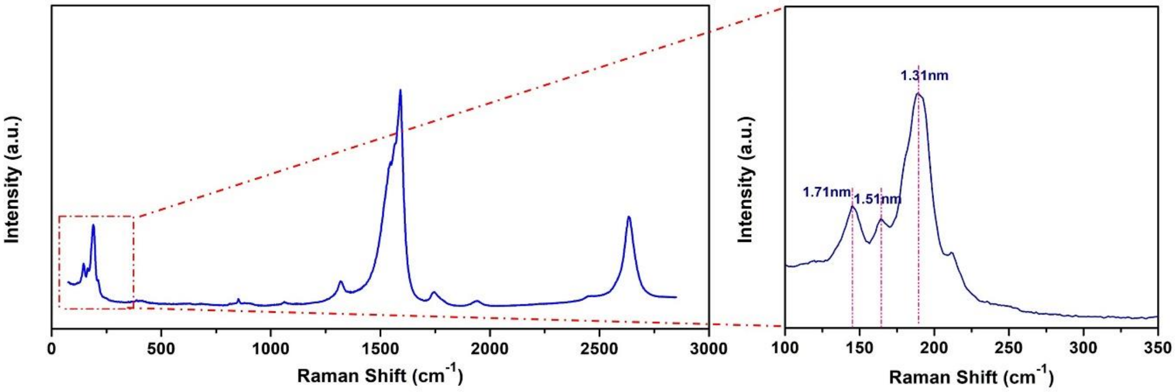

Figure S2. Raman spectra of the SWCNTs used in this work.

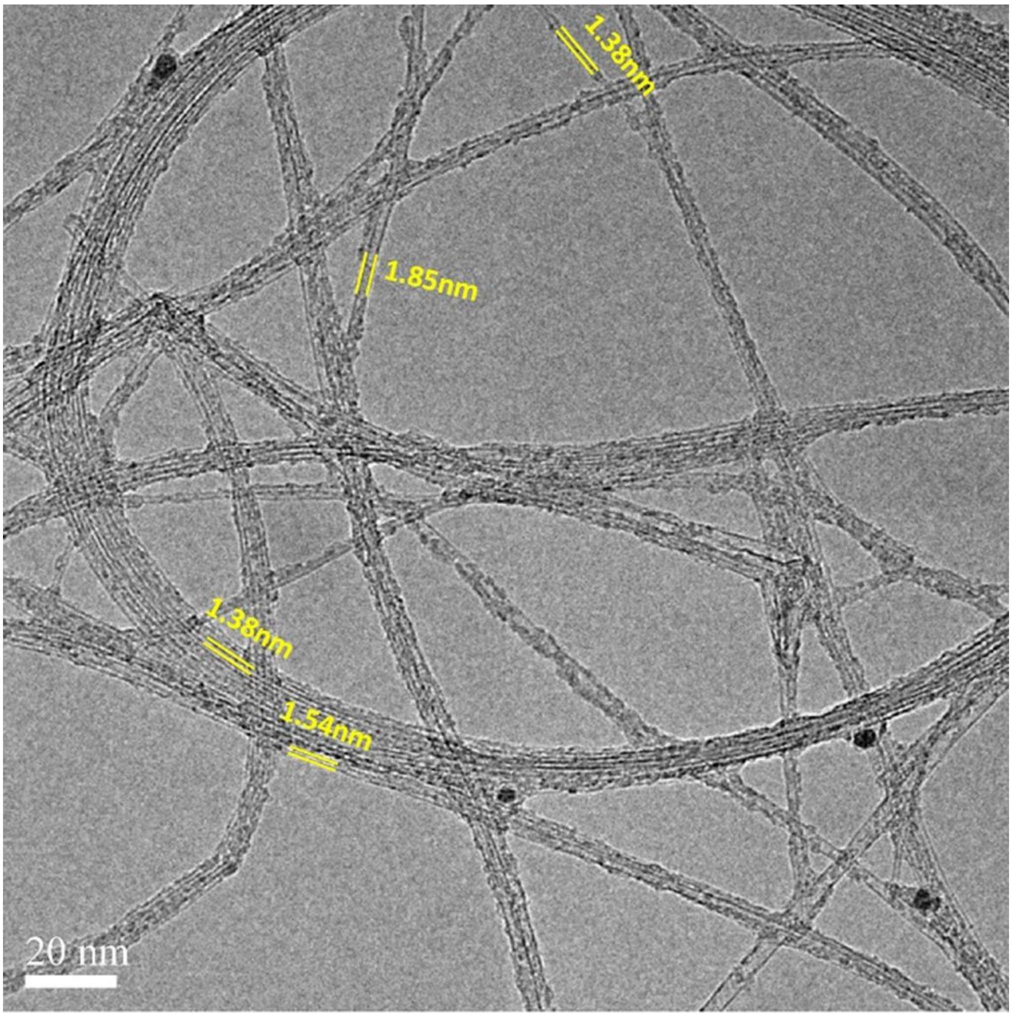

Figure S3. TEM image of the SWCNTs used in this work.

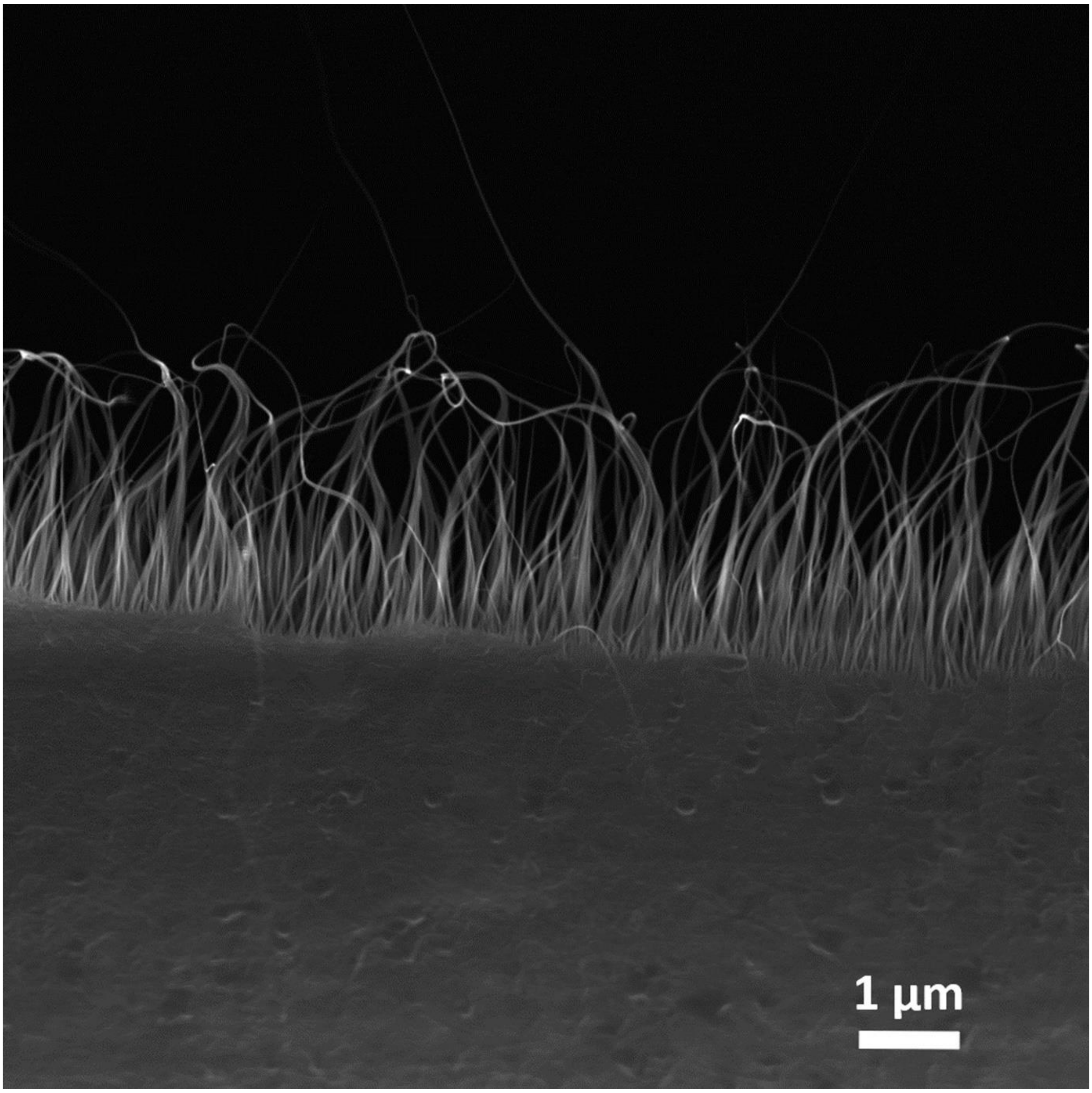

Figure S4. SEM image of the SWCNT film.

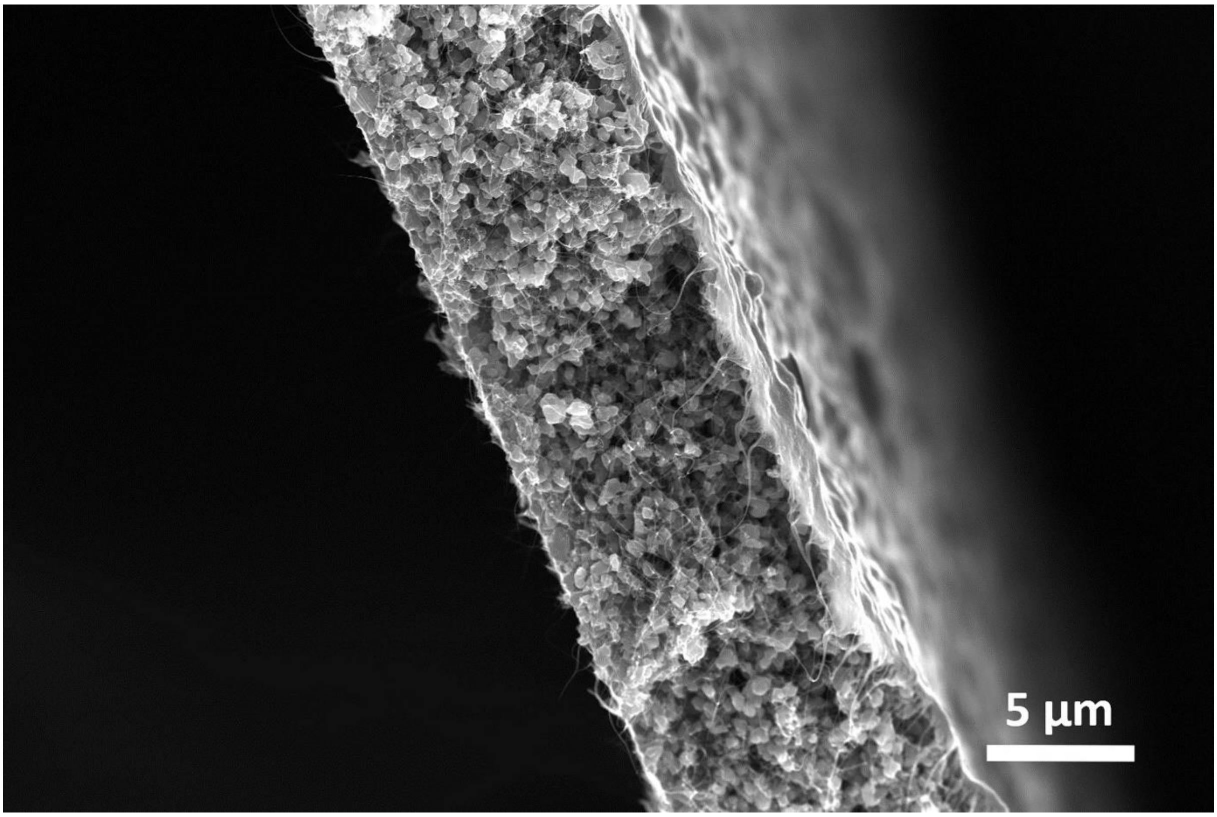

Figure S5. SWCNT film sprayed directly onto the surface of the active materials.

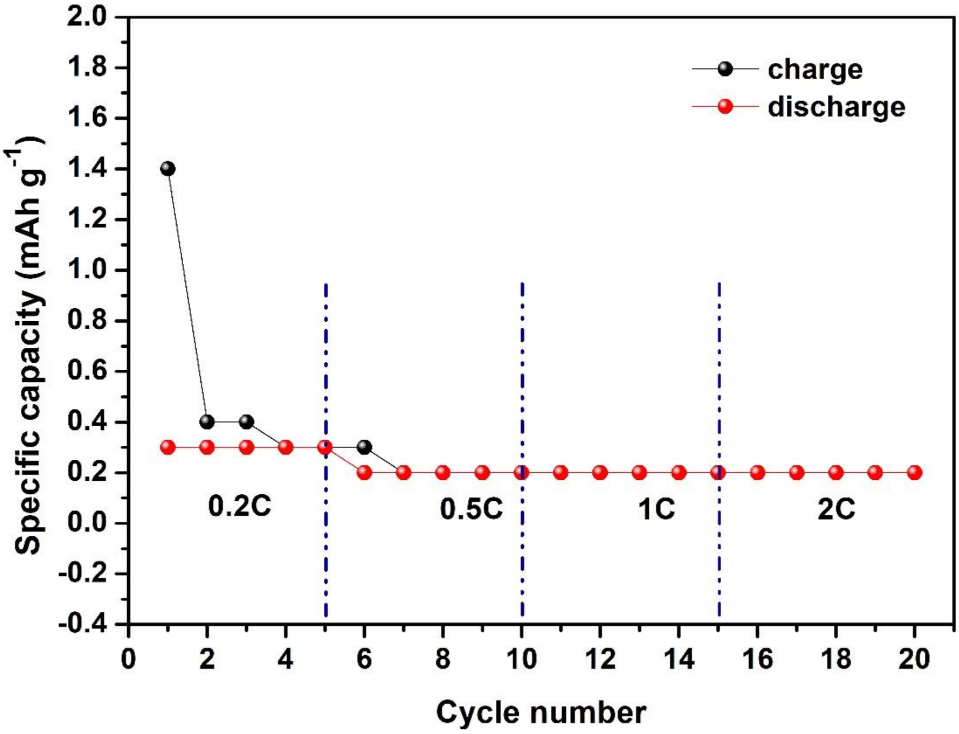

Figure S6. Specific capacity of the SWCNT film in the range 2.5-4.2V.

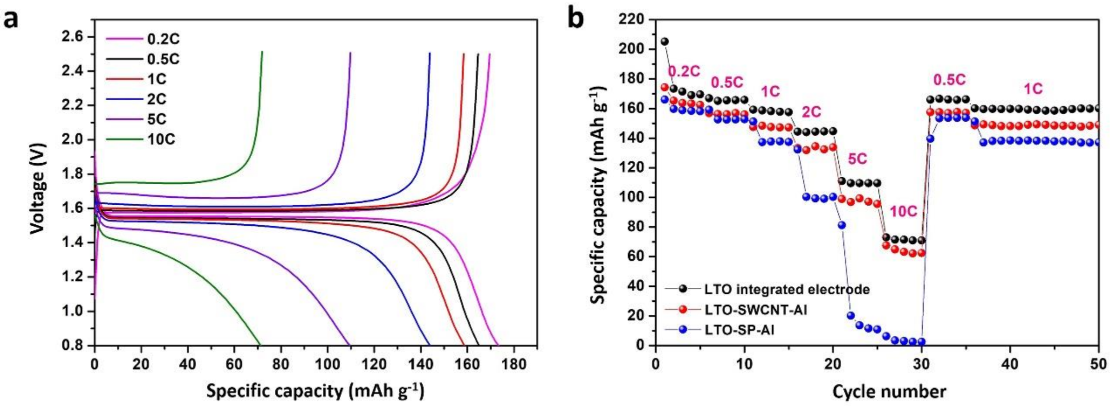

Figure S7. Electrochemical properties of LTO integrated electrodes. a) Charge/discharge curves and b) rate capability at different rates for the flexible integrated electrode, LTO-SWCNT-Al electrode and LTO-Al electrode.

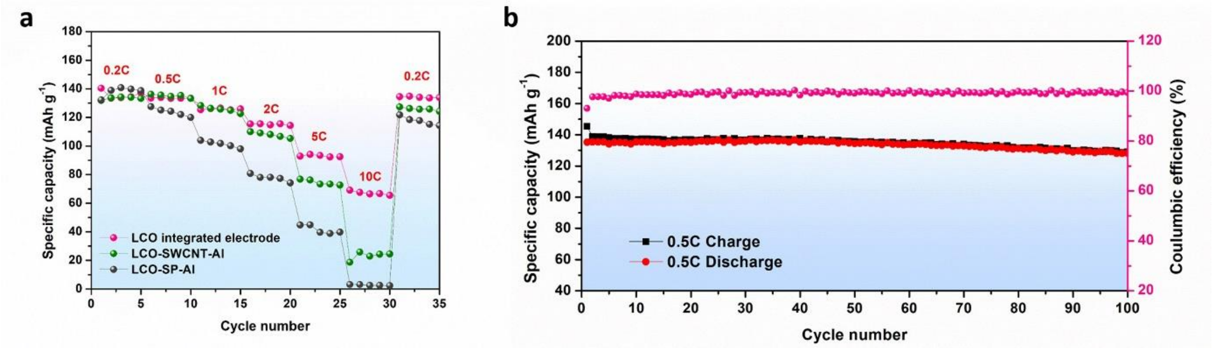

Figure S8. Electrochemical properties of LCO integrated electrodes. a) Rate capability at different rates for the LCO integrated electrode, LCO-SWCNT-Al electrode and LCO-Al electrode. b) Cycling performance of the LCO integrated electrode at 0.5C.

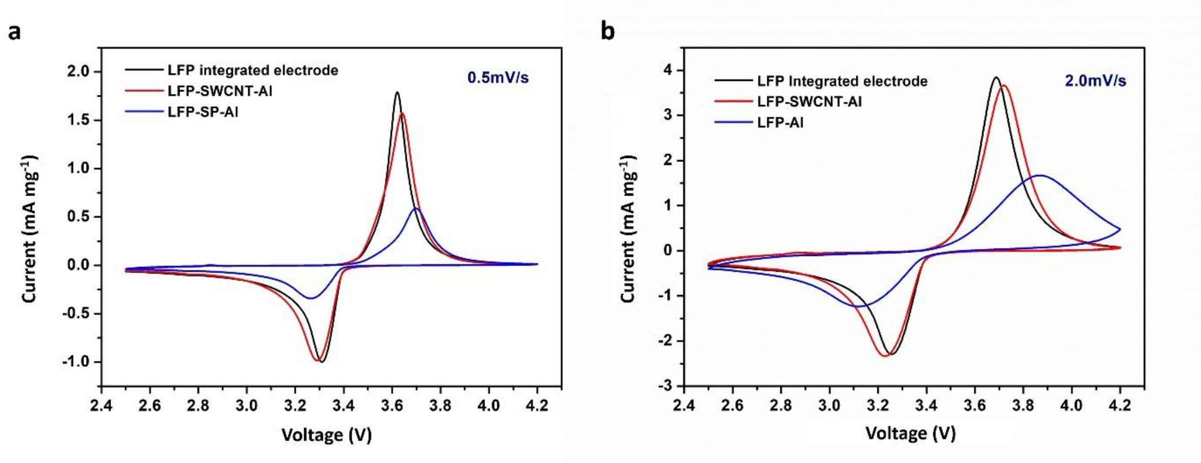

Figure S9. CV curves of the LFP electrodes at a) 0.5 mV s^{-1} and b) 2.0 mV s^{-1}.

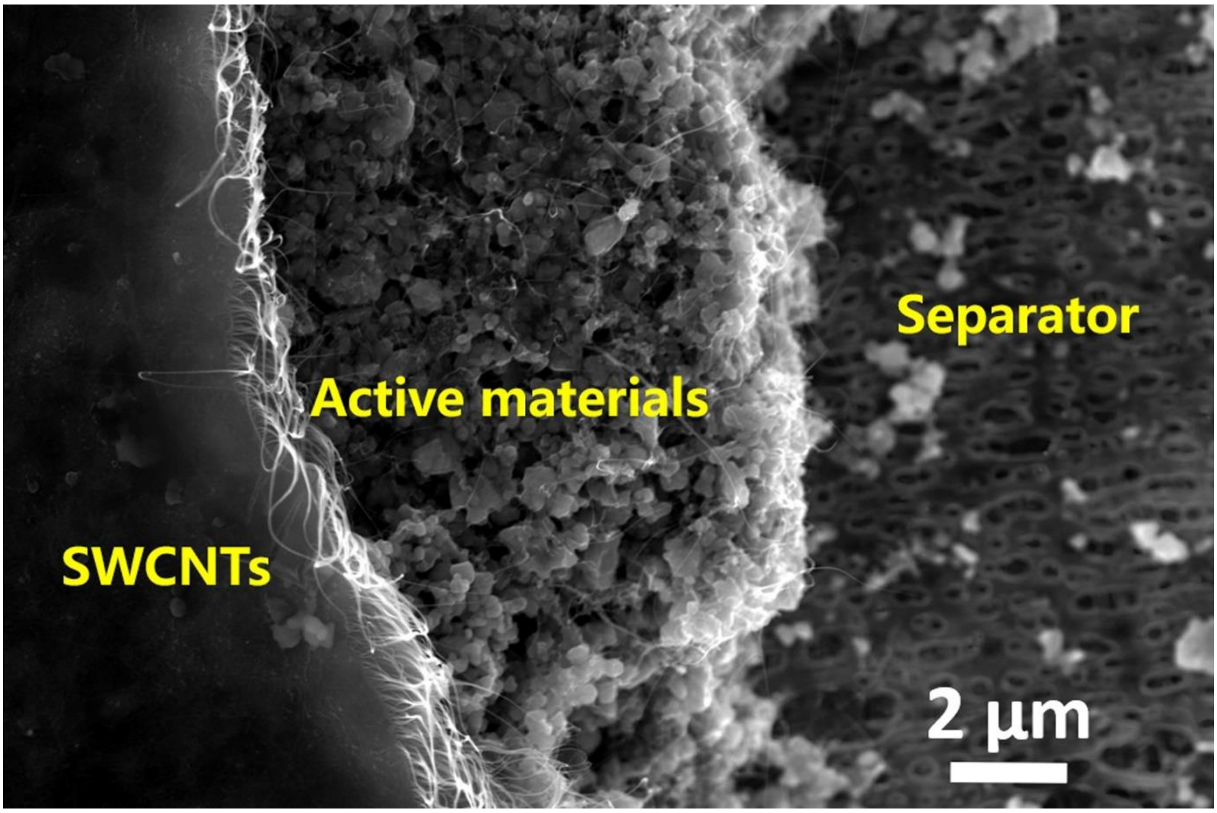

Figure S10. SEM image of a cycled electrode after tape-stripping.

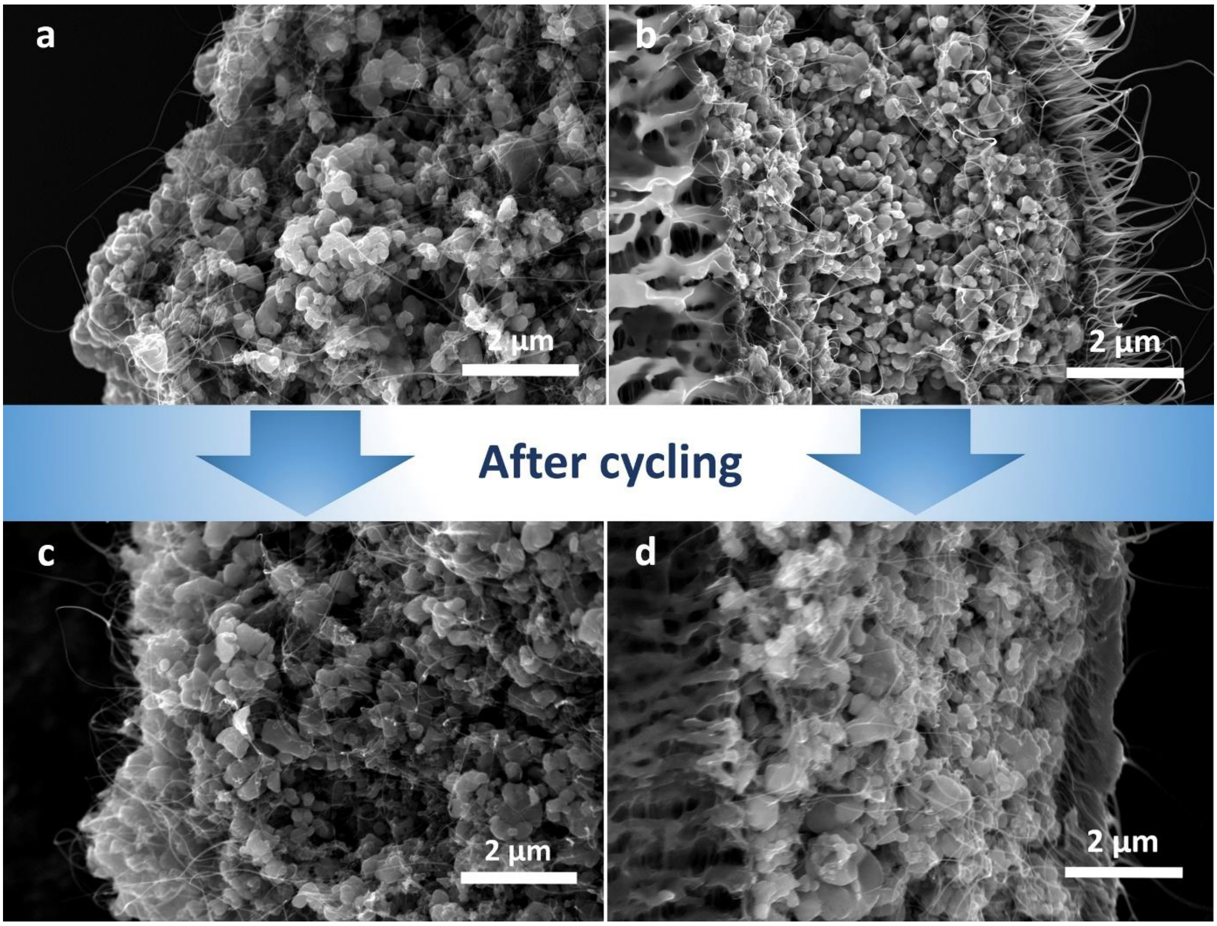

Figure S11. Comparison of the electrode morphology before and after charge/discharge cycles. SEM images for the active materials (LFP) and integrated electrodes a, b) before and c, d) after 1650 cycles.

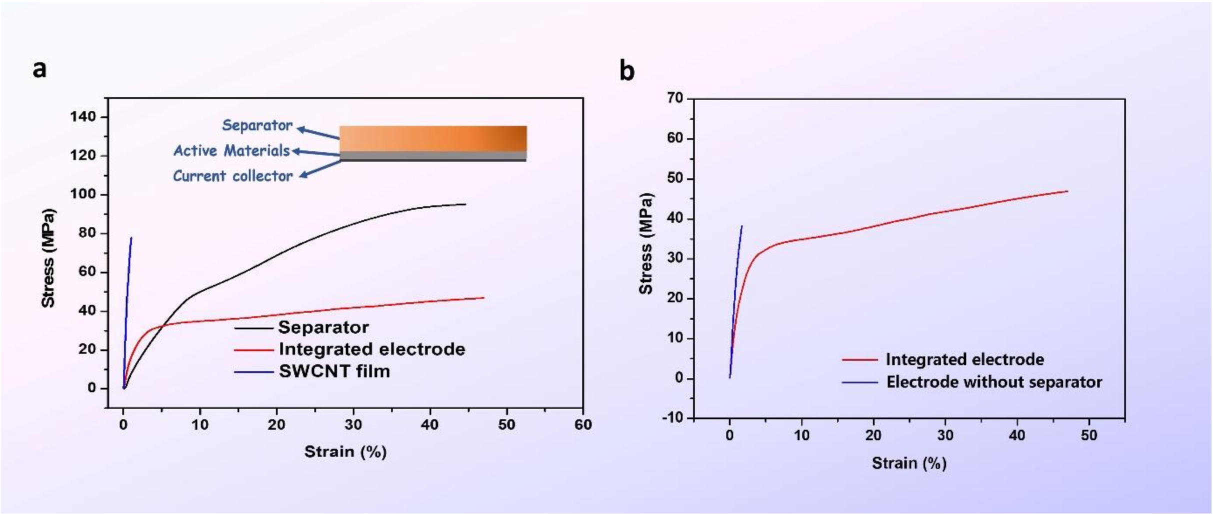

Figure S12. Mechanical properties of integrated electrodes. a) Comparison of the tensile properties of the separator, integrated electrode and SWCNT film. b) Comparison of the mechanical properties of the

integrated electrode and the electrode without a separator as substrate.

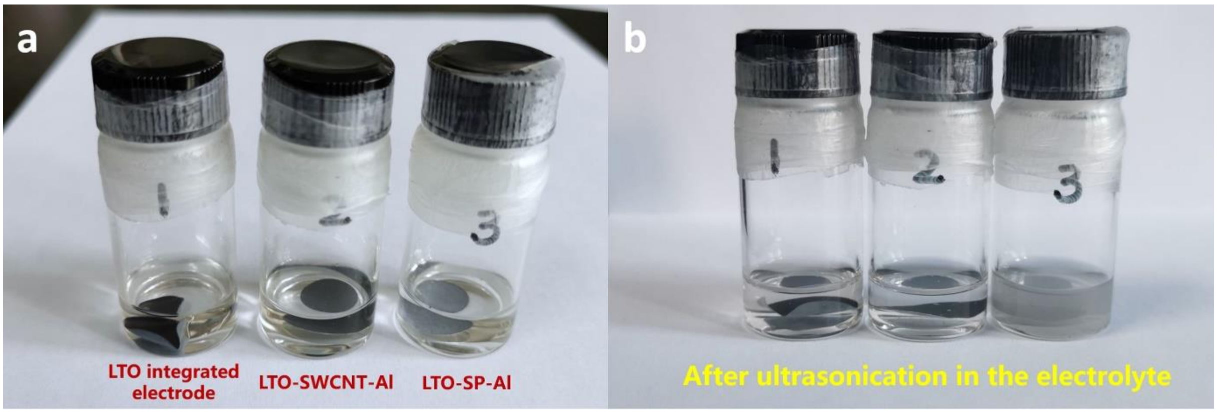

Figure S13. Comparison of the stability of the three electrodes in the electrolyte.

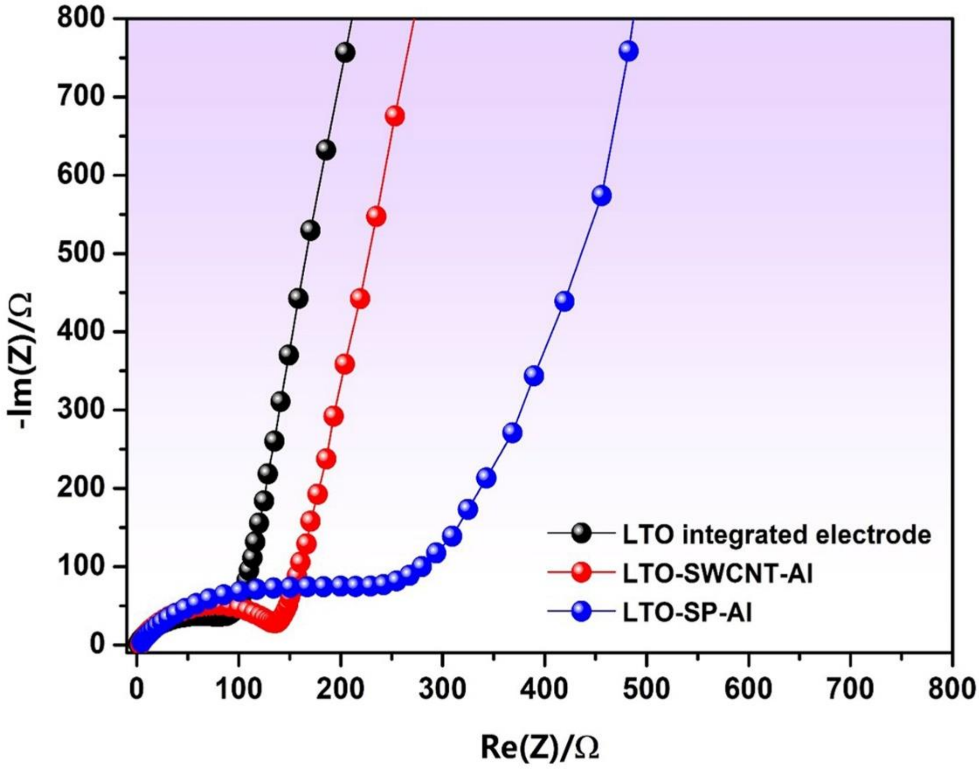

Figure S14. EIS curves for the LTO integrated, LTO-SWCNT-Al and LTO-SP-Al electrodes.

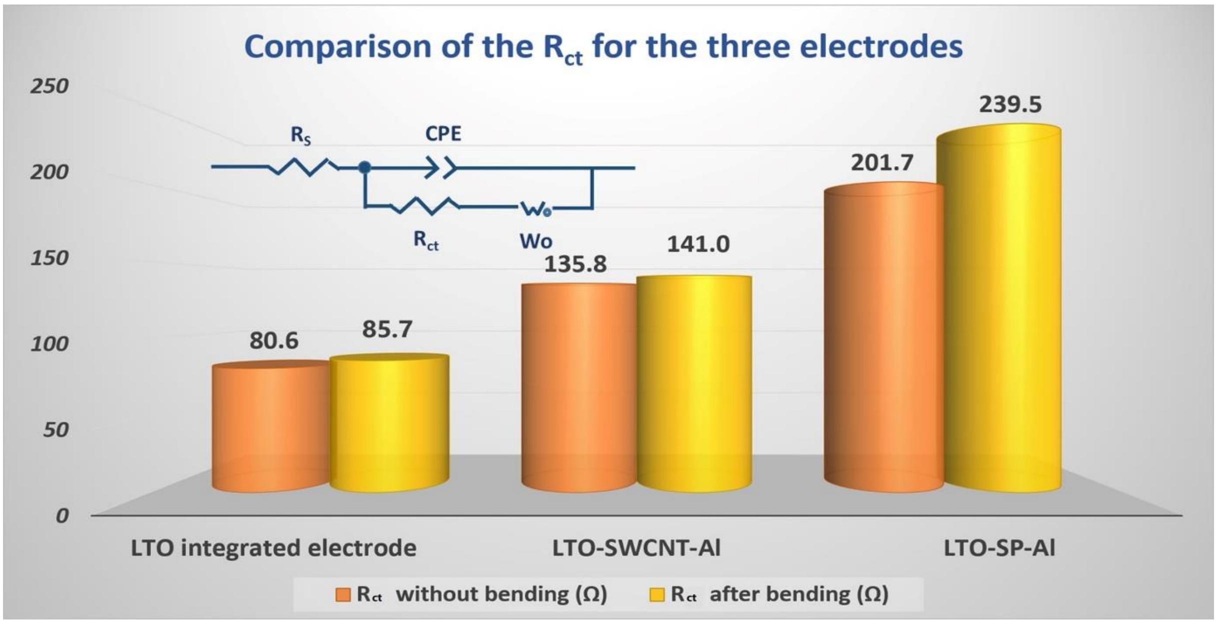

Figure S15. R_{ct} after bending for the three electrodes. Inset is the equivalent circuit corresponding to the EIS results.

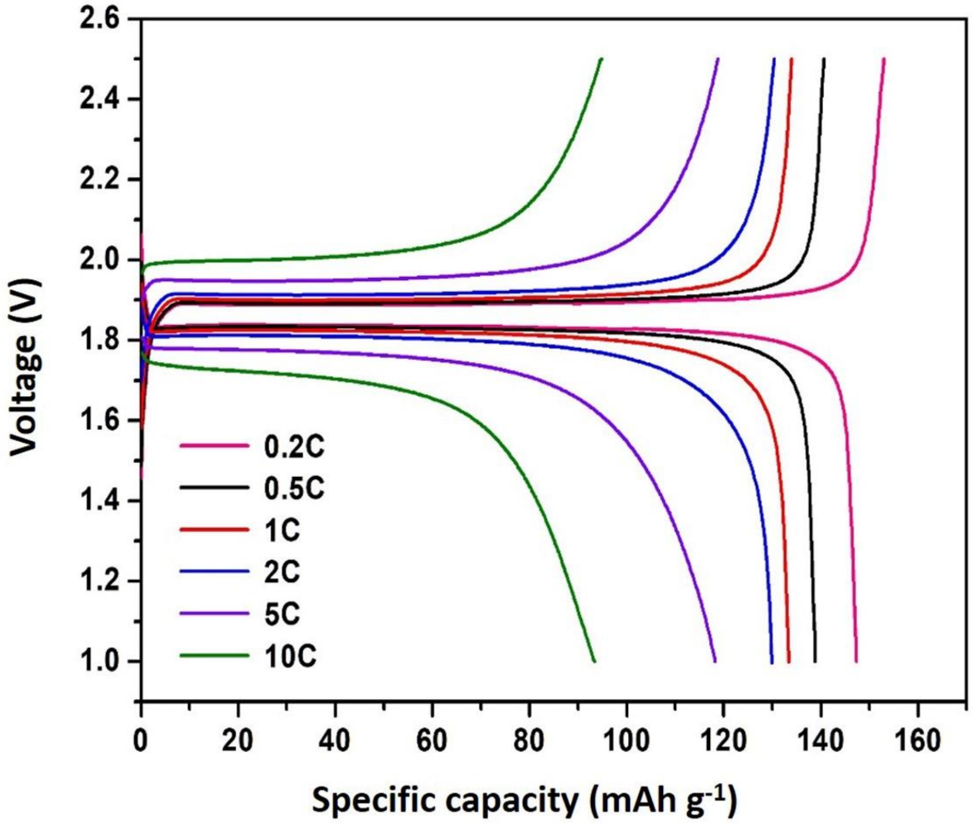

Figure S16. Charge/discharge curves for the full cell at different rates.

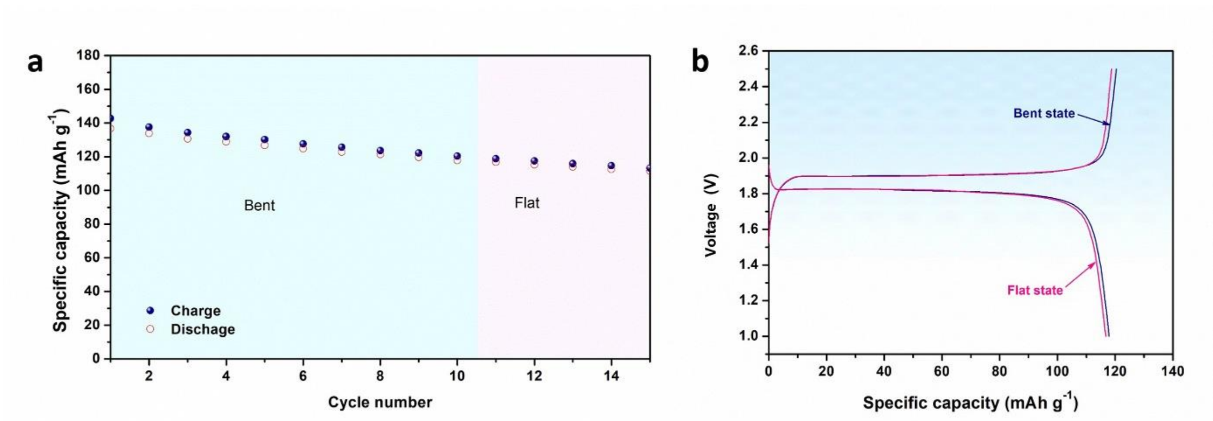

Figure S17. Cycling performance and charge/discharge curves for the full cell at bent and flat state.

Table S1. The comparison of gravimetric energy density of the cathodes

Cathodes with different substrates	Mass percent of LFP in the electrode	C-rate	Specific capacity (mA h g^{-1})	Energy density (Wh kg^{-1})	Ref.
LFP with SWCNT film*	70%	0.2	165.2	367.5	This work
LFP with graphene membrane	~60%	0.5	166	325	[1]
NCM 622 with polymer substrate	43%	0.1	160	249.9	[2]
LFP with TPU	60%	0.2	152	306	[3]
LFP with carbon fabric**	~17.2%	0.25	151	88.5	[4]
LFP ink	33%	0.3	154.5	170.8	[5]
LCO with graphene film	54%-58%	1	150	108	[6]

*The energy density of LFP integrated electrode is calculated with the deduction of the mass of separator to keep the same with other work.

**The density of the carbon fabric is calculated as 1.5g/cm^3.

Table S2. Warburg coefficient (δ) and calculated Li^+ diffusion coefficient (D_{Li+}) from EIS measurements.

Sample	δ (Ω cm^2 $s^{-1/2}$)	D_{Li+} (cm^2 s^{-1})
LTO integrated electrode	66.027	1.98×10^{-13}
LTO-SWCNT-Al	83.01	9.65×10^{-14}
LTO-SP-Al	192.46	1.06×10^{-14}

Movie S1 (.mp4 format). Flexible LIBs with LFP and LTO integrated electrodes during repeated bending.

References

[1] Y. Shi, L. Wen, G. M. Zhou, J. Chen, S. F. Pei, K. Huang, H. M. Cheng, F. Li, *2D Mater.* **2015,** *2*, 024004.

[2] Z. Chen, G. T. Kim, Z. L. Wang, D. Bresser, B. S. Qin, D. Geiger, U. Kaiser, X. S. Wang, Z. X. Shen, S. Passerini, *Nano Energy* **2019,** *64*.

[3] J. J. Bao, B. K. Zou, Q. Cheng, Y. P. Huang, F. Wu, G. W. Xu, C. H. Chen, *J Membrane Sci* **2017,** *541*, 633.

[4] S. H. Ha, K. H. Shin, H. W. Park, Y. J. Lee, *Small* **2018,** *14*.

[5] Y. H. Bao, Y. Liu, Y. D. Kuang, D. N. Fang, T. Li, *Energy Storage Mater* **2020,** *33*.

[6] W. Shen, K. Li, Y. Y. Lv, T. Xu, D. Wei, Z. F. Liu, *Adv. Energy Mater.* **2020,** *10*.
